# Enhanced Osteogenesis of Adipose Derived Stem Cells with Noggin Suppression and Delivery of BMP-2

**DOI:** 10.1371/journal.pone.0072474

**Published:** 2013-08-15

**Authors:** Jiabing Fan, Hyejin Park, Steven Tan, Min Lee

**Affiliations:** 1 Division of Advanced Prosthodontics, University of California Los Angeles, Los Angeles, California, United States of America; 2 Department of Bioengineering, University of California Los Angeles, Los Angeles, California, United States of America; University of California, San Diego, United States of America

## Abstract

Bone morphogenetic proteins (BMPs) are believed to be the most potent osteoinductive factors. However, BMPs are highly pleiotropic molecules and their supra-physiological high dose requirement leads to adverse side effects and inefficient bone formation. Thus, there is a need to develop alternative osteoinductive growth factor strategies that can effectively complement BMP activity. In this study, we intrinsically stimulated BMP signaling in adipose derived stem cells (ASCs) by downregulating noggin, a potent BMP antagonist, using an RNAi strategy. ASCs transduced with noggin shRNA significantly enhanced osteogenic differentiation of cells. The potency of endogenous BMPs was subsequently enhanced by stimulating ASCs with exogenous BMPs at a significantly reduced dose. The level of mineralization in noggin shRNA treated ASCs when treated with BMP-2 was comparable to that of control shRNA treated cell treated with 10-fold more BMP-2. The complementary strategy of noggin suppression + BMP-2 to enhance osteogenesis was further confirmed in 3D *in vitro* environments using scaffolds consisting of chitosan (CH), chondroitin sulfate (CS), and apatite layer on their surfaces designed to slowly release BMP-2. This finding supports the novel therapeutic potential of this complementary strategy in bone regeneration.

## Introduction

Skeletal defects caused by traumas, diseases and accidents remain a major healthcare challenge. Annually, over 1 million skeletal-related treatments occur in the United States [1]. Although autologous bone grafts remain the gold standard for skeletal reconstruction, their applications are limited by donor site availability and associated complications such as donor site morbidity [1].

Tissue engineering approaches are promising alternative strategies to regenerate bone involving sophisticated osteoconductive matrices, osteogenic precursor cells, and appropriate osteoinductive growth factors [2],[3]. In particular, bone morphogenetic proteins (BMP) are considered the most potent osteoinductive factors and have been extensively used in clinical practice [4],[5]. While BMP-2 has demonstrated extraordinary potential in bone formation, their clinical applications require supraphysiological milligram-level doses, leading to undesired ectopic bone and cyst formation [6]–[8]. Additionally, the requirement for high doses of BMP-2 has given patients a high medical burden [9]. Therefore, there is a need to develop alternative strategies that can effectively complement BMP activity.

One alternative approach is to enhance the ability of the progenitor cells that participate in regeneration to respond to endogenous BMPs. In response to BMPs, cells in osteoblastic lineage were found to express BMP antagonists such as noggin in a negative feedback fashion to regulate excessive cellular exposure to BMPs, suggesting that the balance between BMPs and their antagonists is a critical regulator of osteogenesis [10]–[15]. Noggin is a secreted protein that binds to BMP-2, -4, -5, -6, -7 and blocks BMP functions by preventing their binding with BMP receptors [16]–[18]. Several reports have demonstrated that the inhibition of BMP signaling by exogenous application of noggin significantly impaired bone formation both *in vitro* and *in vivo* [19]–[23]. On the other hand, administration of noggin-neutralizing antibodies increased osteogenesis presumably by suppressing production of endogenous noggin and enhancing the anabolic effects of BMPs [24].

The potency of endogenous BMPs can be enhanced by delivering exogenous BMP-2 at a reduced dose. This can be accomplished by delivering BMP-2 using scaffolds designed to slowly release BMPs in a controlled manner [25]. We previously developed rapid biomimetic processing strategies to create a biomimetic apatite layer on the various surfaces of complex three-dimensional scaffolds [26]–[28]. The apatite layer not only enhanced overall osteoinductivity of the scaffold but also presented a sustained release of loaded proteins with reduced burst [26]. Thus, enhancing endogenous BMP activity through the suppression of noggin and the controlled delivery of exogenous BMP-2 may augment bone regeneration and minimize potential adverse effects associated with the application of supraphysiological BMP doses.

This study investigates whether noggin suppression and simultaneous treatment with BMP-2 can enhance osteogenic differentiation of mesenchymal cells using adipose derived stem cells (ASCs). We first downregulated noggin in ASCs using noggin-targeted short hairpin RNA (shRNA) and their osteogenic differentiation was observed with the addition of different concentrations of BMP-2. We further confirmed the complementary strategy to enhance BMP signaling in *in vitro* three-dimensional setting by using scaffolds derived from natural polysaccharides, chitosan (CH) and chondroitin sulfate (CS), with a biomimetic apatite layer on their surfaces to provide a sustained release of BMP-2.

## Materials and Methods

### Ethics statement

All studies involving animal were carried out using protocol approved by the UCLA Animal Research Committee (Approval #2008-073 and #2003-093) and were in compliance with Guidelines for the Care and Use of Laboratory Animal of the National Institutes of Health.

### Isolation of cells

ASCs were harvested from inguinal fat pads of 4∼8 weeks C57BL/6 mice purchased from Charles River Laboratories (Wilmington, MA) as previously described [29]. Briefly, dissected adipose tissues were rinsed with sterilized phosphate-buffered saline (PBS), cut into small pieces, and digested with 0.1% collagenase type I (Sigma, St. Louis, MO) for 2 h. Cells were collected from the digested solution by centrifugation at 1,200 rpm for 5min and washed with sterilized PBS three times. Collected cells were resuspended in a basal culture medium of Dulbeccos’ modified Eagles’ medium (DMEM, Gibco, NY) with low glucose, 10% fetal bovine serum (FBS, Gibco, NY), 1% penicillin/streptomycin (Gibco), and seeded onto tissue culture flasks. To evaluate self-renewal capability of ASCs, the harvested cells at passage 1 were seeded onto 10-cm tissue culture plates at a density of 50 cells/cm^2^. After 14 days of culture, cells were stained with crystal violet solution (Sigma) for 5 min as previously described [30] and the formation of cell colonies was observed by a light microscope. To evaluate the differentiation capacity of ASCs, the harvested cells were cultured in specific differentiation media. To investigate osteogenesis, cells were cultured for 14 days in osteogenic media consisting of DMEM basal medium, 50 µg/mL L-ascorbic acid, 10 mM glycerophosphate, and 100 nM dexamethasone and calcium deposition in cells were detected with Alizarin Red staining. For chondrogenesis, cells were cultured for 21 days in chondrogenic media consisting of DMEM basal medium, 5 µg/ml insulin, 1 mM sodium pyruvate, and 100 nM dexamethasone and deposition of proteoglycan in cells was observed with Safranin-O staining. For adipogenic differentiation, cells were cultured for 14 days in adipogenic media consisting of DMEM basal medium, 5 µg/ml insulin, 0.5 mM 3-isobutyl-1-methylxanthine, 0.1 mM indomethacin, and l µM dexamethasone and lipid accumulation in cells were detected with Oil Red staining. Experiments were performed in triplicate.

### Gene transduction

ASCs were transduced with the lentiviral particles (Santa Cruz, Dallas, TX) containing short hairpin RNA (shRNA) targeting the noggin gene according to manufacturer’s instructions. Briefly, the cells at Passage 3 were seeded onto 6 well plates. When at 50% confluence after 24 h, cells were incubated with the mixture of shRNA lentiviral particles and Polybrene (8µg/ml) overnight. Stable clones expressing shRNA were selected via puromycin dihydrochloride selection. The lentiviral particles encoding a scrambled shRNA sequence were as negative controls. The lentivirus particles expressing eGFP were applied to these experiments. The efficiency of transduction was evaluated using lentiviral particles containing green fluorescence protein (GFP) gene. Suppression of noggin transcript was observed by quantitative real-time PCR.

### Alkaline phosphatase (ALP) activity and Alizarin red staining

ASCs infected with noggin-targeted shRNA and control shRNA were cultured in growth medium consisting of DMEM and 10% FBS. When cells arrived at 90% confluence, the growth medium was changed to an osteogenic medium supplemented with 50 µg/mL L-ascorbic acid, 10 mM β-glycerophosphate, and 100 nM dexamethason (Sigma). ALP staining was performed as previously reported [31]. After 3 days of culture, cells were fixed in 10% formalin, washed with PBS, and incubated in staining solution which was made by mixture of 5-Brom-4-chlor-3-indoxylphosphat (BCIP, Sigma) and Nitro Blue tetrazolium (NBT, Sigma) stock solutions in AP buffer (100 mM Tris pH 8.5, 100 mM NaCl, 50 mM MgCl_2_) for 1 h.

Alizarin red staining was performed as previously reported [31]. After 14 days of culture, cells were fixed in 10% formalin, washed with PBS, and incubated in 2% Alizarin Red staining solution for 5 min. The staining reactions were stopped by washing cells with PBS. The stained samples were then observed with the Olympus BX51 microscope. The blue color indicates expressed ALP and the red color indicates calcium deposition. ALP and mineral production were quantified by image analysis of ALP staining and Alizarin red staining samples. ALP and Alizarin Red staining intensity was quantified by selecting Regions Of Interest (ROI) followed by “integrated density” measurements using ImageJ (NIH, Bethesda, Maryland) as previously described [32]. Briefly, circular ROI were selected, thresholded to measure positively stained areas, then mean gray value (pixel intensity) was determined from multiple images (7–10) to calculate integrated density (mean gray value × area).

### Quantitative real-time polymerase chain reaction (qRT-PCR)

To investigate the effects of noggin knockdown and BMP-2 on differentiation of ASCs, ASCs infected with noggin-targeted shRNA and control shRNA were cultured in osteogenic medium supplemented with various concentrations (0, 10, 30, 60, 100 ng/ml) of recombinant human BMP-2 (R&D Systems, Minneapolis, MN). After 14 days of culture, total RNA was extracted using Trizol reagent and RNeasy Mini Plant kit (Qiagen, Valencia, CA). http://www.sciencedirect.com/science/article/pii/S1742706109003912 - bib27#bib270.5 µg of total RNA was reverse transcribed to cDNA using a high-capacity cDNA transcription kit (Invitrogen, Carlsbad, CA). Quantitative real-time PCR was performed using LightCycler 480 PCR system (Indianapolis, IN) with 20 ul SYBR Green reaction volume. PCR amplification was performed for 45 cycles. The expression of GAPDH was used to normalize gene expression levels. The following primers were used [31] – *Runx2*: CGGTCTCCTTCCAGGATGGT (forward), GCTTCCGTCAGCGTCAACA (reverse); *ALP*: GTTGCCAAGCTGGGAAGAACAC (forward), CCCACCCCGCTATTCCAAAC (reverse); *Osteocalcin*: GGGAGACAACAGGGAGGAAAC (forward), CAGGCTTCCTGCCAGTACCT (reverse); *Collagen1a1*: AACCCGAGGTATGCTTGATCT (forward), CCAGTTCTTCATTGCATTGC (reverse); *Osteonectin* GCCCCTCAGCAGACTGAA (forward), GGTTGGCACCCACAGGTA (reverse); *Noggin*: CCGGGCTTTATGGCTACTTC (forward), TCCAGCCCTTTGATCTCG (reverse); *GAPDH*: AGGTCGGTGTGAACGGATTTG (forward), TGTAGACCATGTAGTTGAGGTCA (reverse). Experiments were performed in triplicate.

### Fabrication of scaffolds

Apatite-coated CH/CS scaffolds were fabricated as previously described [26]. CH (4.5% w/v, 85% deacetylated, Sigma) in 1 N acetic acid was mixed with CS (9% w/v, ≥ 60% type A, Sigma) in PBS at a ratio of 2:1 (v/v) to yield CH/CS mixture (3% CH and 3% CS). The mixtures were homogenized with a mechanical blender, poured into tissue-culture plates, frozen at –80°C, and lyophilized in a freeze dryer. The obtained CH/CS scaffolds were neutralized by immersing them into 1 N NaOH for 1 h and crosslinked with 5% (w/v) pentasodium tripolyphosphate (TPP, Sigma) solution for 30 min. The scaffolds were sterilized by immersing them in 70% ethanol for 30 min and lyophilized. The scaffolds were cut into 1 mm thick sections with 6 mm in diameter.

Simulated body fluids (SBF) solution for coating the surface of scaffolds was prepared as described previously [26], [27]. Briefly, CaCl_2_, MgCl_2_·6H_2_O, NaHCO_3_, and K_2_HPO_4_·3H_2_O was sequentially dissolved into ddH_2_O and the pH of solution was adjusted to pH 6.0 to increase ionic solubility. Na_2_SO_4_, KCl, and NaCl were then added and the final pH of solution was adjusted to pH 6.5 (SBF 1). HCO_3_^−^ free SBF (SBF 2) and Mg^2+^ was prepared by subsequently dissolving NaCl, CaCl_2_, and K_2_HPO_4_·3H_2_O and the pH of solution was adjusted to pH 6.4 to increase ionic solubility. To improve wetting and coating uniformity, the obtained CH/CS scaffolds were subjected to glow discharge argon plasma etching (Harrick Scientific, Ossining, NY). The etched scaffolds were incubated in SBF 1 for 1 day and transferred to SBF 2 for another 2 days at 37°C. The apatite-coated scaffolds were washed with ddH_2_O to get rid of excess ions and lyophilized prior to further study.

The internal morphology of scaffolds was examined by scanning electron microscopy (SEM, Nova Nano SEM 230/FEI, Hillsboro, OR). The cross-sectioned samples were mounted on aluminum stubs and sputter-coated with gold at 20 mA under 70 mTorr for 50 s.

### *In vitro* release of BMP-2

To examine the release kinetics of the protein, rhBMP-2 was absorbed onto the apatite- coated CH/CS scaffolds. Briefly, 30 µL of protein solution in PBS was dropped onto scaffolds at a final protein concentration of 15 µg/ml. The scaffolds were dried for 15 min and further lyophilized in a freeze dryer. The protein-loaded scaffolds were immersed in 1 mL of 10 mM PBS (pH 7.4) at 37°C. The entire incubating solution was removed and replaced with 1 mL of fresh PBS solution at predetermined time points over 21 days. The amount of released protein in the supernatant was determined by a quantikine BMP-2 ELISA kit (R&D system) according to manufacturer’s protocol. Briefly, 50 µL of standard (known concentrations of BMP-2), controls (supernatant from PBS-loaded scaffolds), or experimental (supernatant from BMP-loaded scaffolds) samples was added to each well including 100 µL of assay diluent RD1-19. After 2 h incubation and following wash, 200 µL of BMP-2 conjugate was added to each well. After 2 h incubation and following wash, 200 µL of substrate solution was further added to each well. After 30 min incubation, 50 µL stop solution was added to each well, which was measured using a microplate reader at 450 nm. Experiments were performed in triplicate, and the amount of protein released was expressed as a percentage of the initial amount of protein loaded.

### Cells seeding on scaffolds

ASCs infected with noggin-targeted shRNA and control shRNA were seeded on CH/CS scaffolds loaded with PBS or BMP-2 (15 µg/ml) at a concentration of 2×10^6 ^cells/mL. The cell/scaffold constructs were cultured in complete DMEM supplemented with 10 mM β-glycerol phosphate, 50 µg/mL ascorbic acid and 100 nM dexamethason at 37°C in 5% CO_2_ humidified incubators. To observe the viability of the cells cultured on the scaffolds, the cell/scaffold constructs were washed once with PBS and stained with calcein/ethidium homodimer using a LIVE/DEAD assay kit (Invitrogen) at 37°C for 30 min. Stained samples were observed by Leica SP1 confocal microscopy (Buffalo Grove, IL).

### Gene expression and histological analysis

After 14 days of incubation, total RNA in the cell/scaffold constructs was extracted and reverse transcribed to cDNA as described above. Real-time PCR analysis of *Runx2, ALP, Osteocalcin, Collagen1a1* gene expression was performed with Roche LightCycler 480 PCR system (Indianapolis) as described above. For collagen deposition, the cell/scaffold constructs were cultured and collected at 21 days. The samples were fixed in 10% formalin, embedded in paraffin, and sectioned at 9 µm thickness. The sections were deparaffinized and stained with 0.1% Picrosirius red solution (Polysciences, Inc., Warrington, PA) for 1 h as described previously [33]. Stained samples were observed under a microscope. The newly formed collagen appeared as red. Experiments were performed in triplicate.

### Statistical analysis

Statistical analysis was performed using one way analysis of variances (ANOVA) followed by the Tukey’s post hoc test when more than two groups were compared. Student’s *t* test was used to compare differences between two groups. *p*<0.05 was considered statistically significant.

## Results

### ASC characterization

The self-renewal ability of ASCs was determined by cell colony assay. Formation of cell colonies was observed 14 days after culture (Fig. S1a). To confirm the capacity of ASCs to differentiate into multiple mesenchymal lineages, cells are cultured in specific differentiation media. Deposition of proteoglycan, calcium, and oil was observed as assessed by Safranin-O, Alizarin red, and Oil red staining, respectively, suggesting that cells can differentiate along chondrogenic, osteogenic, and adipogenic lineages (Fig. S1b).

### Noggin knockdown in ASCs

**Figure 1:**
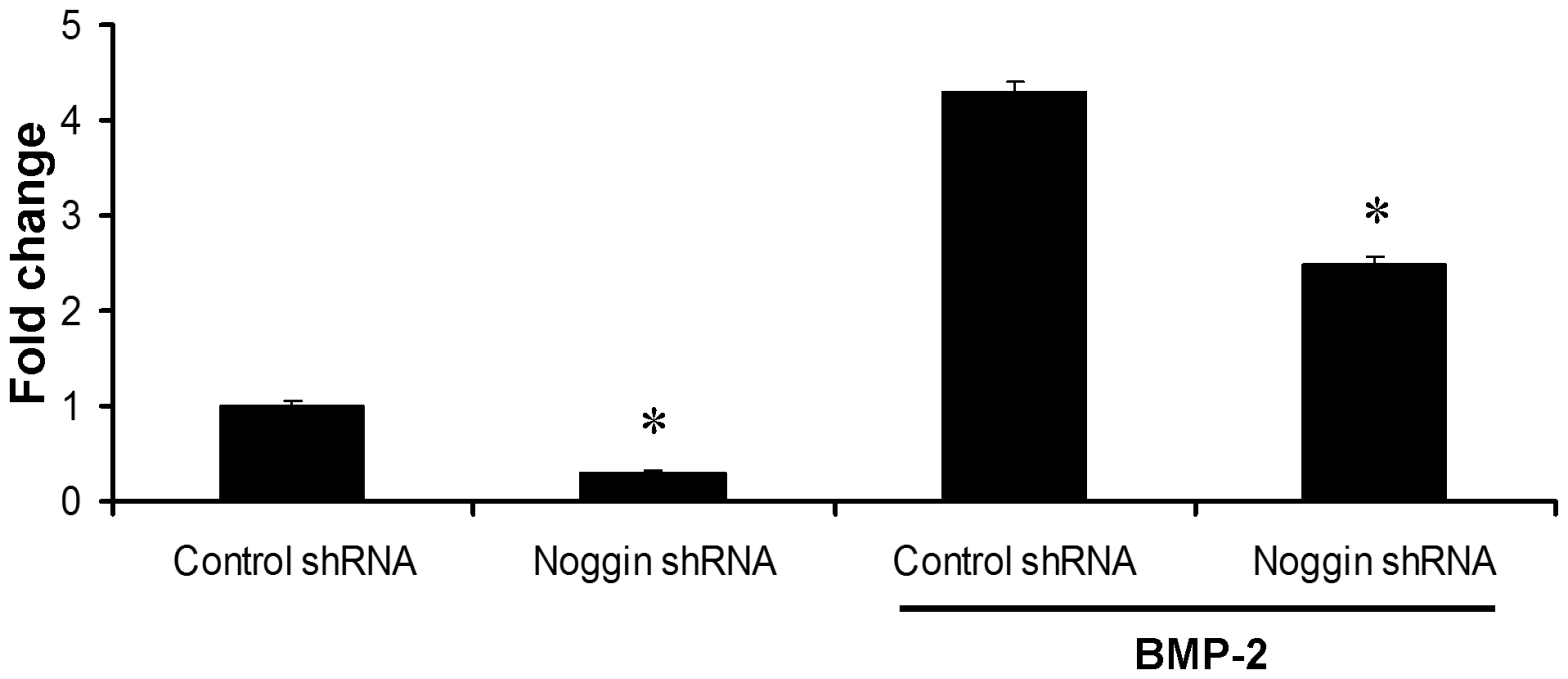
Expressional noggin gene in ASCs transduced with noggin shRNA lentiviral particles. *Noggin* expression was downregulated in ASCs transduced with lentivirus particles targeting noggin shRNA in the presence or absence of BMP-2 (100 ng/ml) as evaluated by quantitative real-time PCR analysis. Significantly lower noggin expression was observed with noggin shRNA transduction (*p<0.05).

Noggin suppression in ASCs was induced using noggin-targeted shRNA lentiviral particles via RNAi-mediated knockdown. qRT-PCR analysis demonstrated that ASCs transduced with noggin-targeted shRNA decreased transcription level of noggin gene to 1.7- and 3.3-fold compared to cells transduced with control shRNA in the presence and absence of BMP-2, respectively (Fig. 1). High transduction efficiency of > 90% was confirmed using lentivirus particles containing a construct encoding enhanced green fluorescent protein (eGFP) (Fig. S2).

### Osteogenesis of ASCs by noggin suppression and BMP-2

**Figure 2:**
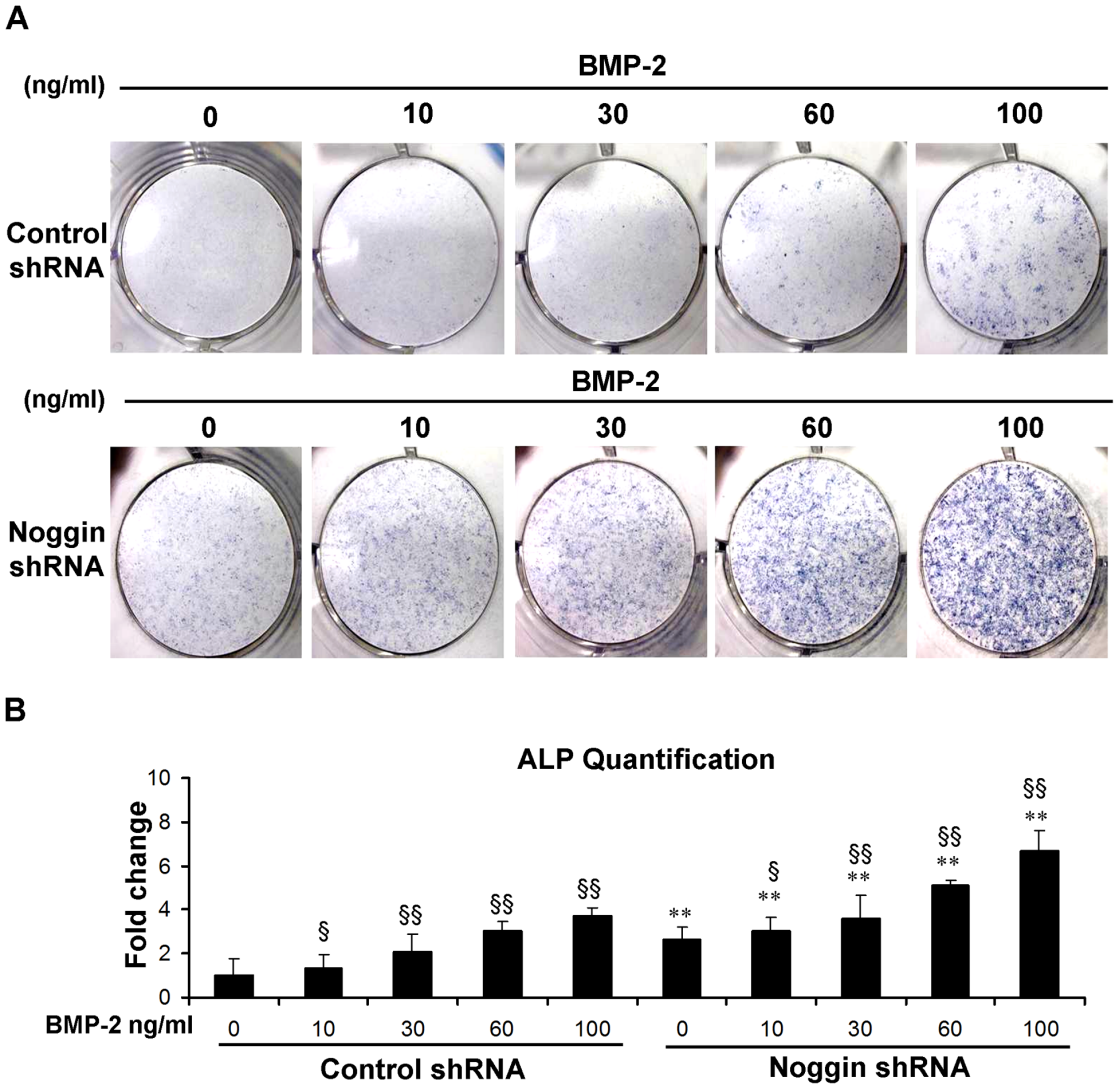
Alkaline phosphatase (ALP) expression of ASCs in monolayer culture. ALP was increased in ASCs transduced with noggin shRNA in the presence or absence of BMP-2 as assessed by ALP staining (A) and quantification (B) at day 3. (**p<0.01 compared to their corresponding controls transduced with control shRNA). Significantly more staining was observed after treatment with different doses of BMP-2 (§ *p* <0.05, §§ *p* <0.01).

Effects of noggin suppression on osteogenic differentiation of ASCs were examined by expression of ALP, an early osteogenic marker, in the presence and absence of BMP-2 (Fig. 2). ALP expression was increased 2.5-fold with noggin shRNA after 3 days of culture. Treatment with BMP-2 increased expression of ALP in control shRNA treated ASCs 1.4- to 3.7-fold as the BMP concentration increased from 10 to 100 ng/ml (Fig. 2b). Noggin suppression further increased ALP expression 6.7-fold with 100 ng/ml BMP-2 treatment, which was significantly higher than that detected in control shRNA treated ASCs (Fig. 2b).

**Figure 3:**
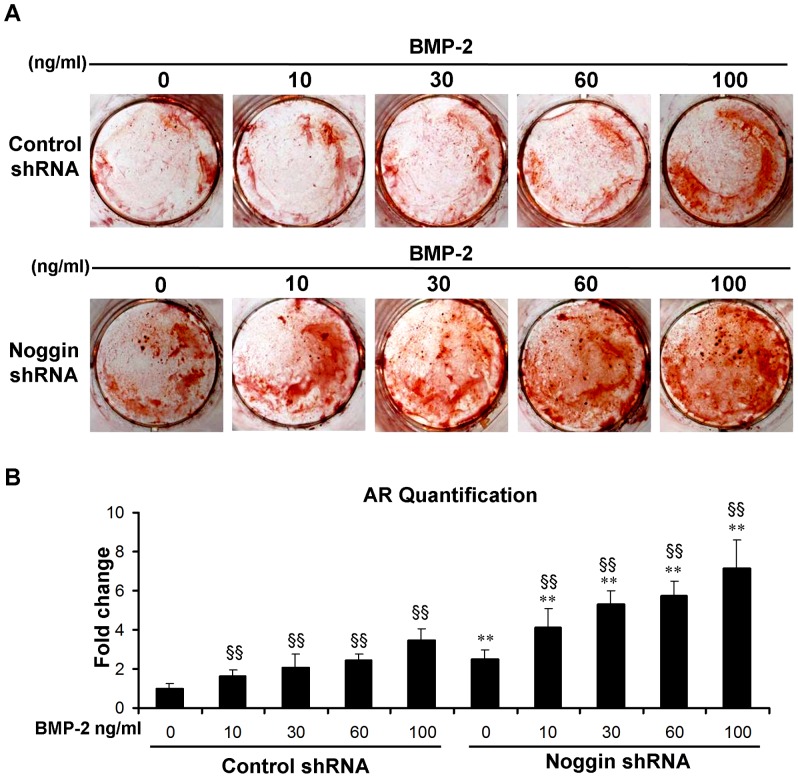
Mineral formation of ASCs in monolayer culture. Mineralization was increased in ASCs transduced with noggin shRNA in the presence or absence of BMP-2 as assessed by Alizarin red staining (A) and quantification (B) at day 14. (**p<0.01 compared to their corresponding controls transduced with control shRNA). Significantly more staining was observed after treatment with different doses of BMP-2 (§§ *p* <0.01).

End stage osteogenesis was further characterized by examining extracelluar matrix mineralization through Alizarin red staining on day 14 (Fig. 3). Extent of mineralization was increased 2.3-fold with noggin suppression in the absence of BMP-2 (Fig. 3b). BMP-2 treatment (from 10 to 100 ng/ml) dose-dependently enhanced mineralization in control shRNA treated ASCs 1.6- to 3.4-fold, which was further increased 4.1- to 7.1-fold with noggin suppression compared with control cells (Fig. 3b). Stimulation of noggin shRNA treated ASCs with 10 ng/ml BMP-2 resulted in mineralization comparable to that detected in control shRNA treated ASCs stimulated with 100 ng/ml BMP-2 (Fig. 3b).

**Figure 4:**
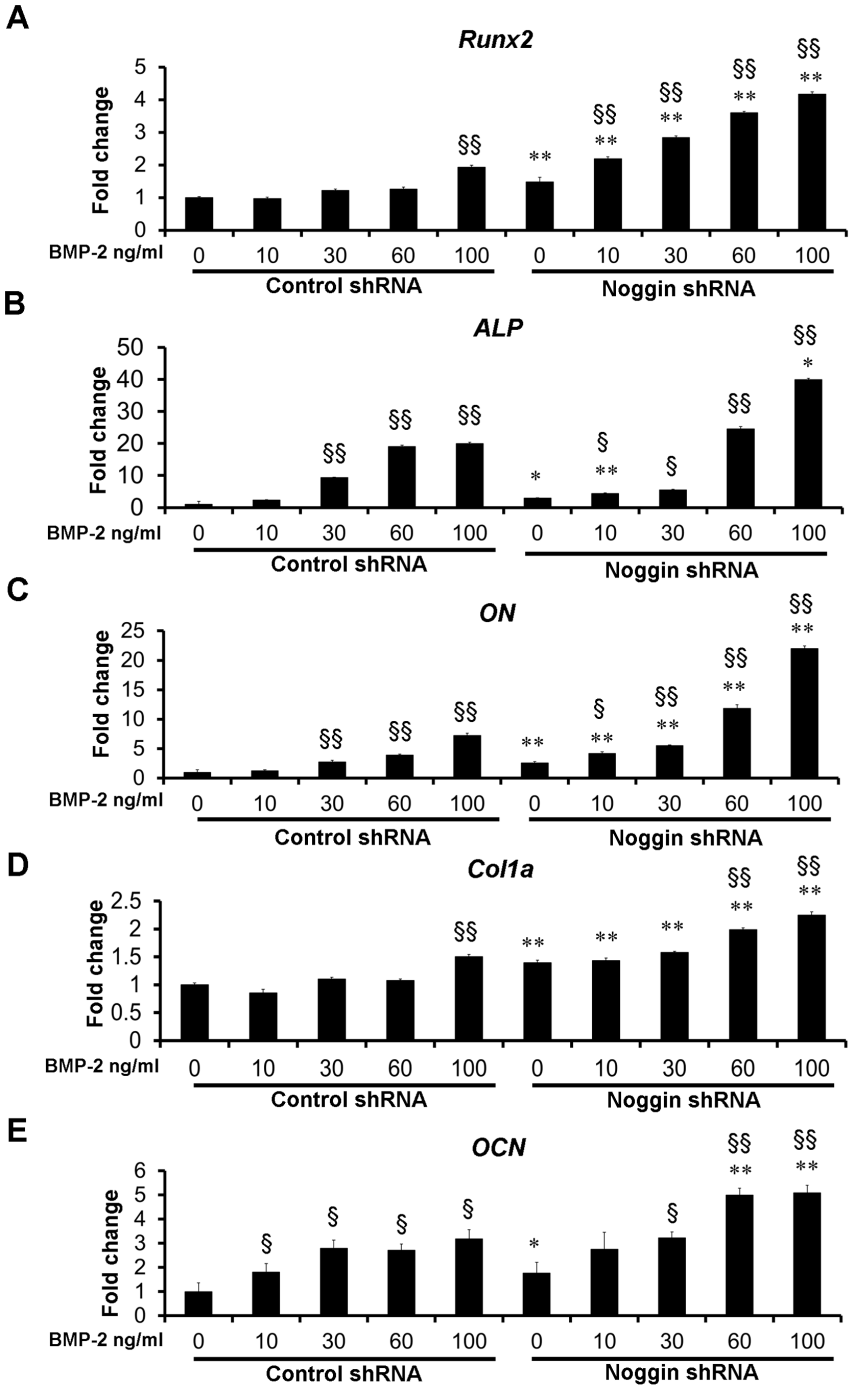
Osteogenic gene expression of ASCs in monolayer culture. Osteogenic gene markers including *Runx2* (A), *ALP* (B), *ON* (C), *Col1a* (D) and *OCN* (E), were elevated in ASCs transduced with noggin shRNA in the presence or absence of BMP-2 as analyzed by quantitative real-time PCR at day 14 (*p<0.05, ** *p*<0.01 compared to their corresponding controls transduced with control shRNA). Significant up-regulation of all genes was observed after treatment with different doses of BMP-2 (§ *p* <0.05, §§ *p* <0.01).

The expression of osteogenic differentiation markers including *Runx2*, *ALP*, *Osteonectin* (*ON*), *Collagen 1a1* (*Col1a*) and *Osteocalcin* (*OCN*) examined by qRT-PCR (Fig. 4). Noggin shRNA transduction increased *ALP* and *ON* expression 2.9- and 2.5-fold, respectively, compared to control shRNA transduction (Fig. 4b, c). When stimulated with various concentrations of BMP-2, the expression of *ALP* and *ON* increased 2.3 to 39.9 fold and 1.3- to 22-fold, respectively, compared with non-BMP treated cells (Fig. 4b, c).

The expression of *Runx2*, *Col1a* and *OCN* was significantly increased by noggin suppression (Fig. 4a, d, e). When stimulated with 30 ng/ml BMP-2, the expression level of *Runx2*, *Col1a* and *OCN* in noggin shRNA treated ASCs was equal or higher than that detected in control shRNA treated ASCs stimulated with 100 ng/ml BMP-2 (Fig. 4a, d, e).

### ASC culture on scaffolds releasing BMP-2

**Figure 5:**
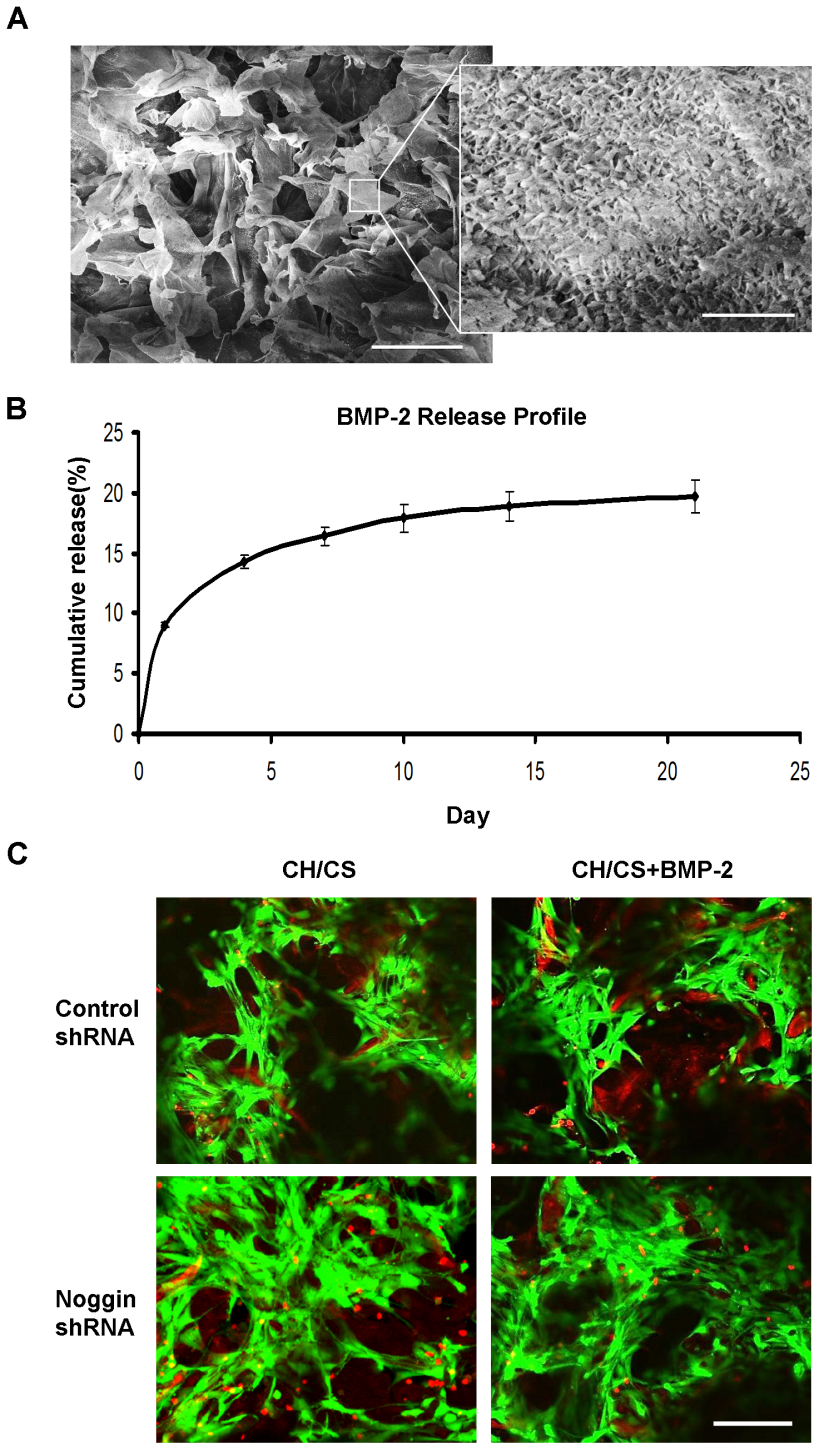
(A) SEM images of apatite-coated CH/CS scaffolds. Scale bar  =  200 µm; scale bar of the inset image  =  20 µm. (B) *In vitro* release of BMP-2 from CH/CS scaffolds. (C) Live-dead fluorescent staining of ASCs seeded on CH/CS scaffolds after 14 days in culture. Scale bar  =  200 µm.

To confirm the osteogenic effects of noggin suppression and BMP treatment in three-dimensional (3D) environments, we developed 3D composite scaffolds containing CH and CS, naturally derived polysaccharides, and biomimetic apatite layer on their surfaces. The prepared scaffolds showed highly interconnected porous structures with a pore size range of 100–150 µm (Fig. 5a).

Apatite coating of the composite scaffolds was achieved by incubating scaffolds in simulated body fluids as described in our previous studies [26]. A uniform layer of apatite coating was created on the surface and the created coating exhibited plate-like morphology (Fig. 5a). The apatite coating did not significantly change the pore size and porosity of the scaffolds [26].

The release kinetics of BMP-2 from the scaffolds was investigated by incubating the scaffolds in PBS (Fig. 5b). ELISA assay showed that approximately 9% of initially loaded BMP-2 was released at day 1, followed by slow release at approximately 0.55% per day (82.5 ng/ml per day) up to day 21.

To investigate the feasibility of these scaffolds to support cell growth, control or noggin shRNA treated ASCs were seeded on the scaffolds loaded with PBS or BMP-2, and observed with Live/Dead staining by confocal microscopy (Fig. 5c). Control shRNA treated ASCs showed viability of 92.0±0.7% and 93.2±1.8% in the scaffolds loaded with PBS and BMP-2, respectively. Noggin shRNA treated ASCs had a similar viability of 91.6±0.6% and 92.8±1.1%.

qRT-PCR analysis demonstrated that ASCs seeded on scaffolds releasing BMP-2 significantly increased the expression level of osteogenic differentiation markers including *Runx2*, *ALP* and *OCN* compared with that detected in control scaffolds.

### Osteogenesis of ASCs by noggin suppression and BMP-2 delivery

**Figure 6:**
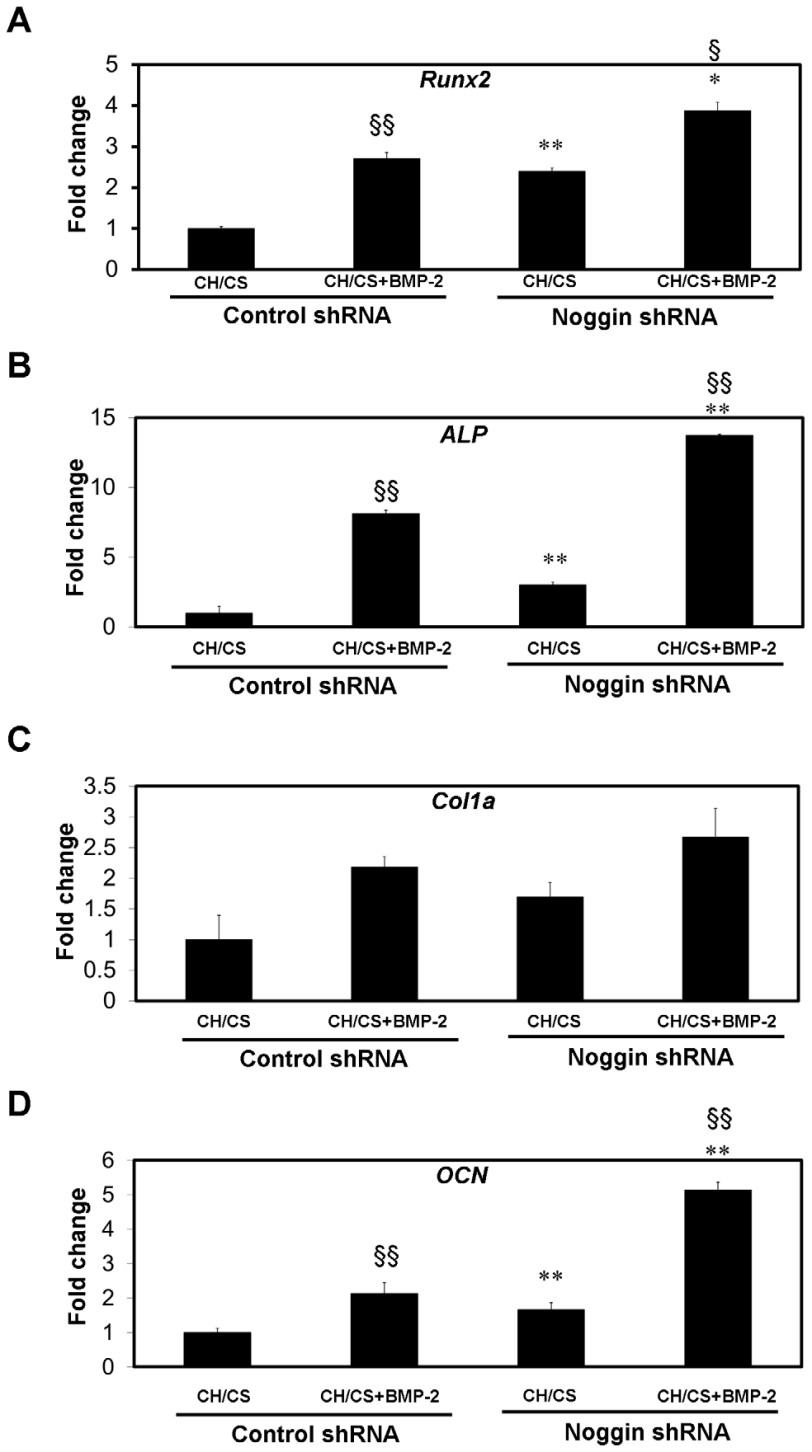
Osteogenic gene expression of ASCs cultured in apatite-coated CH/CS scaffolds releasing BMP-2. Osteogenic gene markers including *Runx2* (A), *ALP* (B), *Col1a* (C) and *OCN* (D) were evaluated in noggin suppressed ASCs cultured on CH/CS scaffolds for 14 days as assessed by quantitative real-time PCR. (*p<0.05, **p<0.01 compared to their corresponding controls transduced with control shRNA). Significant up-regulation of genes including *Runx2*, *ALP* and *OCN* was observed in ASCs cultured on CH/CS scaffolds releasing BMP-2 (§ *p* <0.05, §§ *p* <0.01).

Osteogenic differentiation of ASCs cultured on apatite-coated CH/CS scaffolds was determined by qRT-PCR analysis (Fig. 6). Expression of osteogenic gene markers including *Runx2*, *ALP* and *OCN* were significantly higher in noggin shRNA treated ASCs than that of control shRNA treated ASCs. The osteogenic gene markers in the noggin shRNA treated ASCs were further up-regulated when cultured on the apatite-coated CH/CS scaffolds releasing BMP-2 compared with those of cells cultured on BMP-free control scaffolds (Fig. 6)

**Figure 7:**
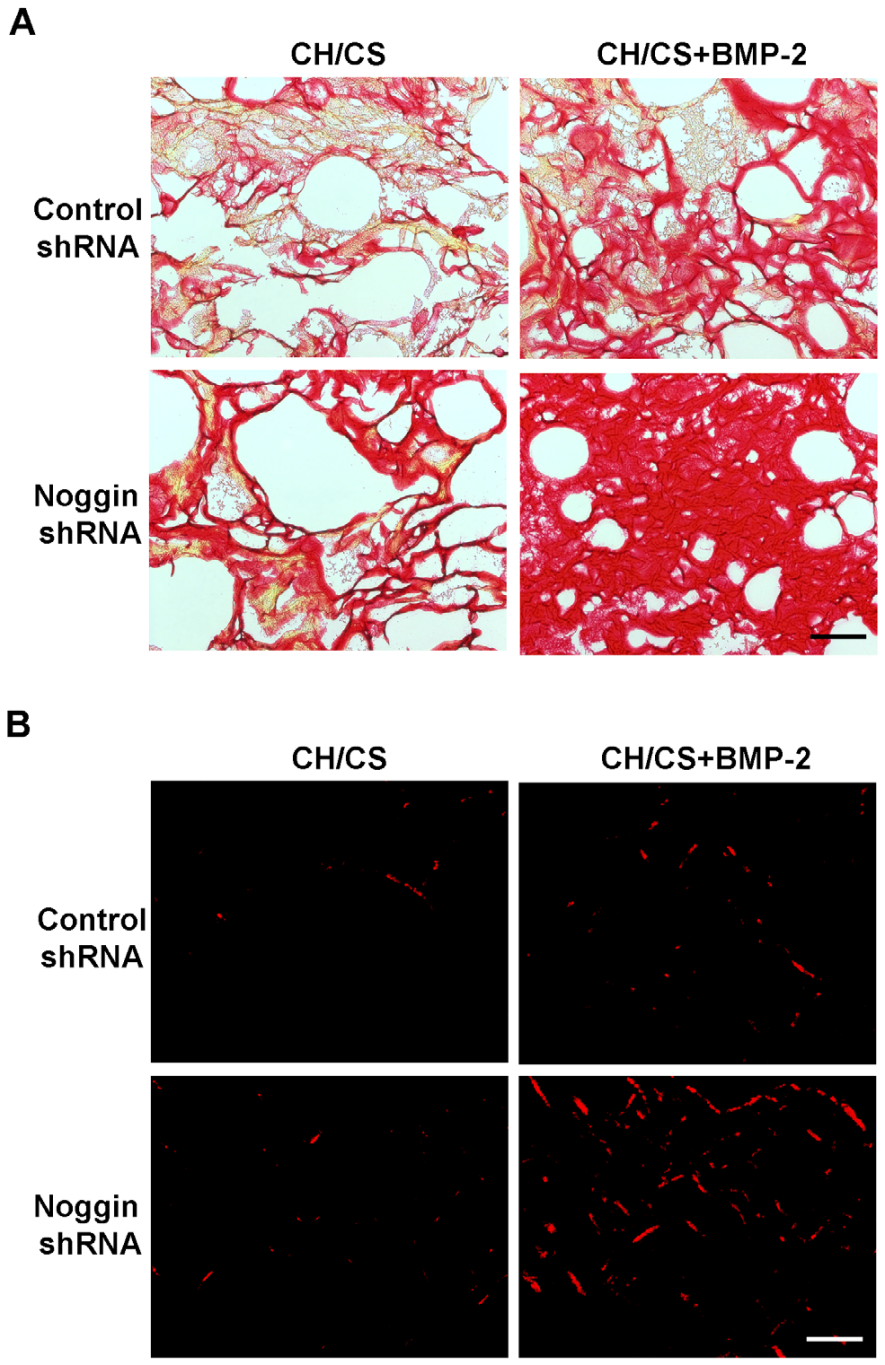
Collagen deposition of ASCs cultured in apatite-coated CH/CS scaffolds releasing BMP-2. Picrosirius red staining of ASCs cultured on CH/CS scaffolds for 21 days, which was assessed by bright field (A) and polarized light (B) microscopy. Scale bar  =  100 µm. Significantly higher collagen deposition was observed in noggin suppressed ASCs cultured on CH/CS scaffolds releasing BMP-2.

Total collagen expression on scaffolds was confirmed by Picrosirius red staining (Fig. 7). The extent of collagen deposition was significantly elevated in noggin shRNA treated ASCs cultured on apatite-coated CH/CS scaffolds releasing BMP-2, compared with control shRNA treated cells cultured on BMP-loaded scaffolds or noggin shRNA treated cells cultured on BMP-free control scaffolds.

## Discussion

To reduce the total dose requirement and minimize potential adverse effects of current osteoinductive therapeutics, we attempted to enhance BMP-induced bone regeneration activity by regulating levels of BMP antagonists while simultaneously delivering BMP-2 in a sustained manner. Noggin is a specific antagonist of BMPs and prevents their interactions with BMP receptors to regulate increased levels of BMP stimuli [11]. Thus, endogenous BMP signaling can be enhanced through by down-regulating Noggin. In addition, endogenous BMPs likely exist as heterodimeric molecules that are reported to be more resistant to Noggin inhibition and more potent in inducing bone formation than their respective homodimers [34]–[36]. Direct therapeutic application of BMP-2 will not be effective in clinical conditions due to the short half-life of the protein and insufficient local concentration at the therapeutic site. Although BMP-2 was often used in combination with a collagen sponge which is soaked with protein solution prior to implantation, it is not an efficient delivery matrix for the local delivery of large amount of BMP-2 for a prolonged period of time [37]. Thus, the need for appropriate carriers that release biological molecules over time in a controlled manner remains critical.

This study demonstrated that transduction of ASCs with noggin shRNA significantly enhanced osteogenic differentiation of cells as shown by upregulation of osteogenic gene expression and mineral formation, indicating enhanced endogenous BMP action by noggin suppression. Stimulation of ASCs with BMP-2 enhanced osteogenic differentiation that was significantly higher in noggin shRNA treated cells compared with control cells, indicating that noggin suppression can enhance activity of exogenous BMPs. The level of osteogenic differentiation in noggin shRNA treated cells treated with BMP-2 (10 ng/ml) was comparable to that of control cells treated with 10-fold more BMP-2 (100 ng/ml) as shown by mineral formation through Alizarin red staining. These results suggest that knock down of noggin expression can significantly reduce the exogenous BMP-2 doses required for effective therapeutic results without compromising efficacy.

With our controlled release platform, we further confirmed our combinatorial strategy of noggin suppression + BMP-2 to enhance osteogenesis in 3D *in vitro* setting using scaffolds designed to slowly release BMP-2. CH and CS used in this study are naturally derived, biocompatible polysaccharides used for many pharmaceutical and biomedical applications. In addition to their excellent biocompatibility, CH and CS are degraded *in vivo* by enzymatic hydrolysis, which makes them attractive as materials for tissue engineering scaffolds [26], [27], [38]. The highly hydrophilic nature of CH with structural similarity to ECM components has been reported to support osteoblast attachment and proliferation [26], [27].

We have extensively investigated cell-scaffold and protein-scaffold interactions in our previous study [26]. Although the hydrophilic nature of CH and structural similarity to ECM components favored osteoblast attachment and proliferation, pure CH scaffolds did not have sufficient mechanical strength. Given that CH with its cationic nature readily forms stable ionic complexes with various anionic molecules, we enhanced the mechanical properties of CH scaffolds by complexing CH with natural anionic polymers such as alginate and CS. Because scaffold materials may influence the attachment and proliferation of progenitor cells, final release kinetics of growth factors, and also biomineralization, we investigated how such additional materials affect cellular responses, biomolecular releasing, and apatite formation on the CH scaffolds. Among the experimental groups, CH/CS composite scaffolds were the most promising to increase the mechanical strength of scaffolds, sustain protein release, enhance apatite layer formation, and support cell growth.

In this study, the fabricated CH/CS scaffold was coated with biomimetic apatite using SBF immersion approach as previously described [26]. The biomimetic apatite coating has been suggested to provide a favorable substrate not only for cell adhesion but also for a sustained release of loaded osteogenic proteins with reduced initial burst on the various biomaterial surfaces, such as Poly Lactic-co-Glycolic-Acid (PLGA), chitosan, tricalcium phosphate (TCP) [26], [39]–[41]. Our study showed that loaded BMP-2 was slowly released in a sustainable manner with approximately 9% of initial burst from apatite-coated CH/CS scaffolds over a 21-day period. The observed sustained release can be attributed to the apatite layers created on the scaffolds providing high protein binding affinity and increased protein retention capacity. Similar to the observation in 2D culture, noggin shRNA treated ASCs seeded on BMP-loaded scaffolds induced more considerable osteogenesis than other groups of scaffolds loaded with BMP-2 or control cells alone.

Our complementary strategy has the advantage of reducing BMP dose by downregulating potent secreted BMP antagonists (such as noggin) while simultaneously delivering BMP-2 at a significantly reduced dose in a sustained pattern. This strategy would significantly improve the clinical efficacy, lower dose requirements, and minimize potential adverse effects of current BMP therapeutics. More work is needed to determine if our complementary strategy can effectively enhance bone formation *in vivo*.

## Conclusions

In this study, we investigated the novel strategy of noggin suppression and BMP delivery to enhance osteogenic differentiation. Knock down of noggin expression significantly reduced the exogenous BMP-2 doses required for effective osteoblastic differentiation. With our controlled release platform, we created scaffolds releasing BMP-2 that significantly enhanced osteogenesis of noggin suppressed cells. These results indicate that noggin suppression combined with delivery of BMP-2 may be a potential therapeutic strategy to promote bone formation by increasing BMP activity while reducing dose requirements.

## Supporting Information

Figure S1 **ASCs proliferation and differentiation ability.** (A) Colony formation assay. ASCs were stained with crystal violet. (B) Multilineage differentiation potential. Chondro-, osteo- and adipogenic differentiation of ASCs were detected with Safranin-O (B1), Alizarin red (B2), and Oil red (B3) staining. Scale bar  =  200 µm.
(TIF)

Figure S2 **Transduction efficiency in ASCs by lentiviral particles.** ASCs were treated with GFP-expressing lentiviral particles and observed under fluorescent microscope. Scale bar  =  100 µm.
(TIF)
