# Supplementary figures and images for: Enhanced Osteogenesis of Adipose Derived Stem Cells with Noggin Suppression and Delivery of BMP-2
Source: PLoS One. 2013 Aug 15;8(8):e72474. doi: 10.1371/journal.pone.0072474 (PMC3744499; doi:10.1371/journal.pone.0072474)

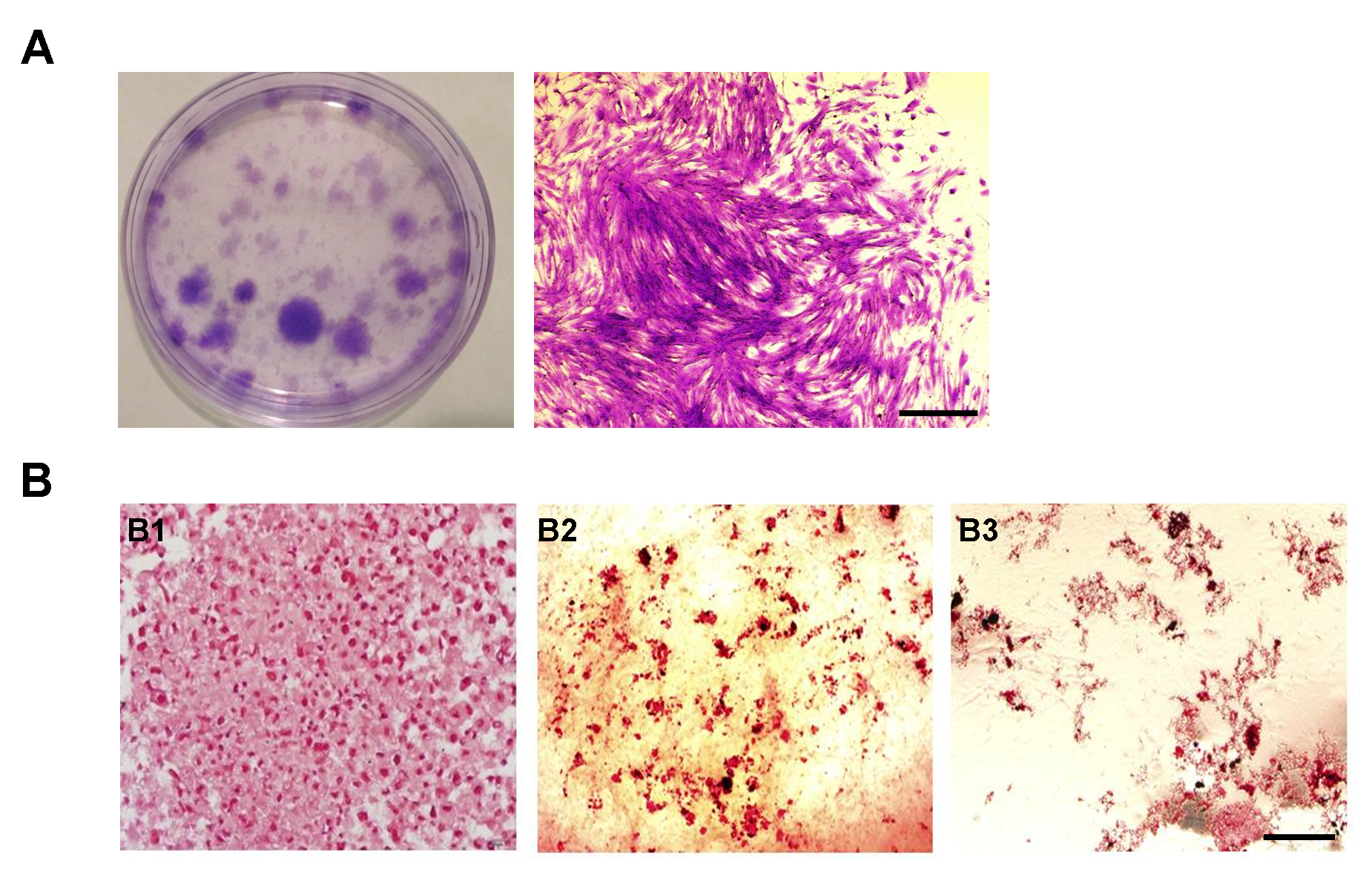

Supplement: Figure S1 — ASCs proliferation and differentiation ability. (A) Colony formation assay. ASCs were stained with crystal violet. (B) Multilineage differentiation potential. Chondro-, osteo- and adipogenic differentiation of ASCs were detected with Safranin-O (B1), Alizarin red (B2), and Oil red (B3) staining. Scale bar = 200 µm. (TIF) [file pone.0072474.s001.tif]

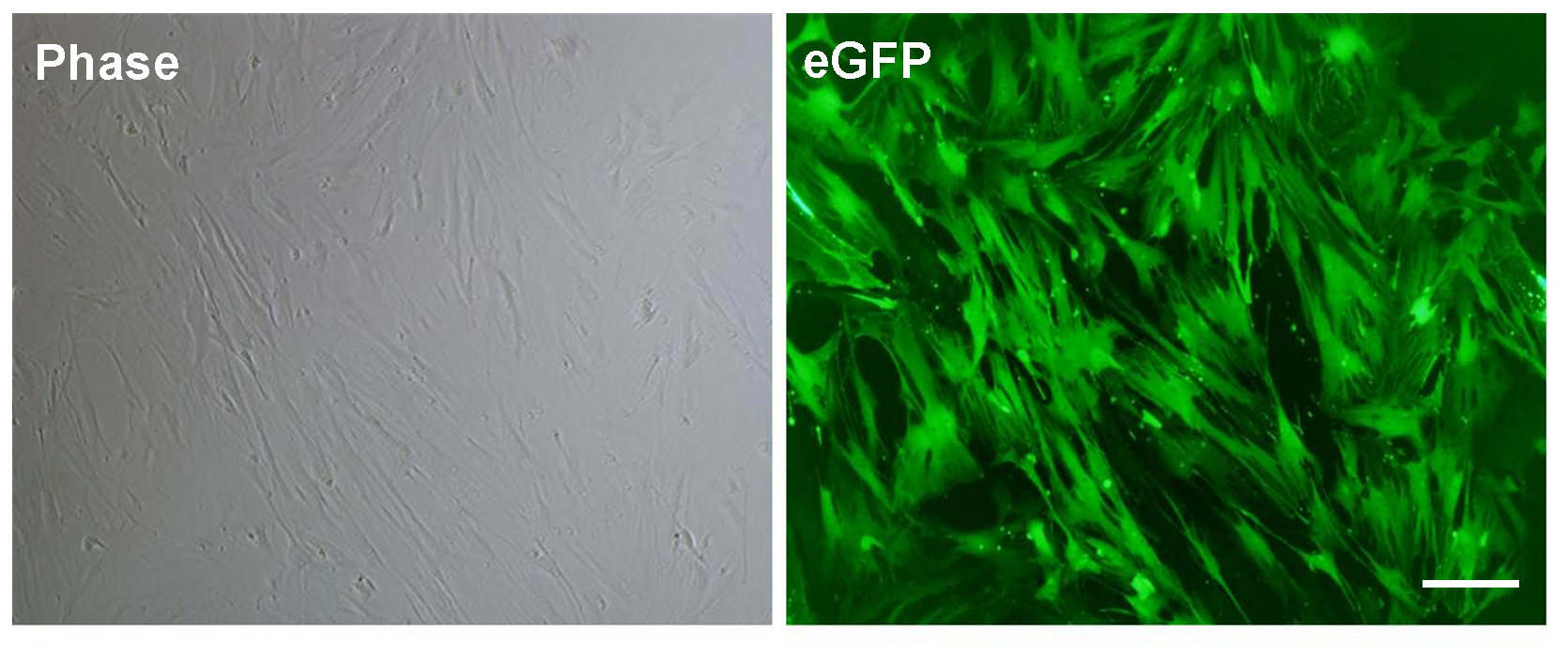

Supplement: Figure S2 — Transduction efficiency in ASCs by lentiviral particles. ASCs were treated with GFP-expressing lentiviral particles and observed under fluorescent microscope. Scale bar = 100 µm. (TIF) [file pone.0072474.s002.tif]
